# Supplementary material for: Amount and intensity of physical activity and risk of incident cancer in the UK Biobank
Source: medRxiv. 2023 Dec 4:2023.12.04.23299386. Preprint. [Version 1] doi: 10.1101/2023.12.04.23299386 (PMC10760289; doi:10.1101/2023.12.04.23299386)
Supplement: Supplement 1 [file NIHPP2023.12.04.23299386v1-supplement-1.pdf]

eTABLES

eTable 1: Definition of variables from the UK Biobank data.
eTable 2. Coding and number of site-specific cancer incident cases in UK Biobank participants.
eTable 3. Tabulation of number of incident primary events per cancer site in 86 556 UK Biobank participants.
eTable 4. Strengthening the Reporting of Observational Studies in Epidemiology (STROBE) statement.
eTable 5. Sequential model adjustments for total physical activity (milligravity units) and risk of incident cancer in 86 556 UK Biobank participants.
eTable 6. Sequential model adjustments for quintile of total physical activity (milligravity units), quintile of median daily step count, and risk of incident physical-activity-related cancer in 86 556 UK Biobank participants.
eTable 7. Changes in behaviour time and physical-activity-related cancer risk in 86 556 UK Biobank participants.
eTable 8. Sequential model adjustments for median daily step count and physical-activity-related cancer risk in 86 556 UK Biobank participants.
eTable 9. Sequential model adjustments for total physical activity (milligravity units) and risk of incident cancer in the UK Biobank among 38 078 male UK Biobank participants.
eTable 10. Sequential model adjustments for total physical activity (milligravity units) and risk of incident cancer in the UK Biobank among 48 478 female UK Biobank participants.
eTable 11. Sex-specific adjusted hazard ratios for median daily step count and physical-activity-related cancer risk in 38 078 male UK Biobank participants and 48 478 female UK Biobank participants.
eTable 12. Models for quintile of total physical activity (milligravity units), quintile of median daily step count, and risk of incident physical-activity-related cancer in UK Biobank participants who were never smokers (N=49 736).
eTable 13. Models for quintile of total physical activity (milligravity units), quintile of median daily step count, and risk of incident physical-activity-related cancer in 86 556 UK Biobank participants before and after removing the first two years of follow-up (N=83 435).

eFIGURES

eFigure 1. Participant flow diagram for the analysis of daily physical activity and step count measured by accelerometers in UK Biobank participants.
eFigure 2. Hazard Ratios reallocating time to a given movement behaviour from all other behaviours proportionally and incident physical-activity-related cancer in 86 556 UK Biobank participants.
eFigure 3. Hazard ratios for all behaviour pairs and incident physical-activity-related cancer risk for estimated using a multivariable-adjusted Cox regression model in 86 556 UK Biobank participants before (blue) and after adjusting for body mass index as a sensitivity analysis.
eFigure 4. Hazard ratios for all behaviour pairs and incident physical-activity-related cancer risk for estimated using a multivariable-adjusted Cox regression model in 86 556 UK Biobank participants before (blue) and after adjusting for dietary factors as a sensitivity analysis.
eFigure 5. Hazard ratios for all behaviour pairs and incident physical-activity-related cancer risk for estimated using a multivariable-adjusted Cox regression model among 48 478 female participants (blue) and 38 078 male participants (red).
eFigure 6. Hazard ratios for all behaviour pairs and incident physical-activity-related cancer risk for estimated using a multivariable-adjusted Cox regression model in 86 556 UK Biobank participants before (blue) and after restricting to never smokers (red, N=49 736).
eFigure 7. Hazard ratios for all behaviour pairs and incident physical-activity-related cancer risk for estimated using a multivariable-adjusted Cox regression model in 86 556 UK Biobank participants before (blue) and after removing the first two years of follow-up (red, N=83 435).

eTable 1. Definition of variables from the UK Biobank data.

Characteristic	Source	Notes	UK Biobank field	Coding Notes
OUTCOME				
Age at first cancer event	Cancer Registry, Death Registry	First ICD-10 code in cancer registry or death registry data. Full list of ICD-10 and ICD-O-3 codes are described in detail in eTable 1.	Derived from 100092	
Age at loss-to-follow up	Death Registry		Derived from 100093	
EXPOSURE				
Total physical activity	Accelerometry	Derived using the Biobank Accelerometer Analysis Tool, “accelerometer”, version 7.1.0. Details: https://github.com/OxWearables/biobankAccelerometerAnalysis		
Step count	Accelerometry	Derived using the “stepcount” tool, version 3.1.1. Details can be found at: https://github.com/OxWearables/stepcount		
Peak 30 second cadence	Accelerometry	Derived using the “stepcount” tool, version 3.1.1. Details can be found at: https://github.com/OxWearables/stepcount		
Sleep	Accelerometry	Derived using the Biobank Accelerometer Analysis Tool, “accelerometer”, version 7.1.0. Details: https://github.com/OxWearables/biobankAccelerometerAnalysis		
Sedentary behaviour	Accelerometry	Derived using the Biobank Accelerometer Analysis Tool, “accelerometer”, version 7.1.0. Details: https://github.com/OxWearables/biobankAccelerometerAnalysis		
Light intensity physical activity behaviour	Accelerometry	Derived using the Biobank Accelerometer Analysis Tool, “accelerometer”, version 7.1.0. Details: https://github.com/OxWearables/biobankAccelerometerAnalysis		

Characteristic	Source	Notes	UK Biobank field	Coding Notes
Moderate-vigorous intensity physical activity behaviours	Accelerometry	Derived using the Biobank Accelerometer Analysis Tool, “accelerometer”, version 7.1.0. Details: https://github.com/OxWearables/biobankAccelerometerAnalysis		
EXCLUSION VARIABLES – MAIN ANALYSIS				
Prior incident cancer	Cancer Registry	Cancer-related ICD-10 codes in cancer registry data prior to accelerometry wear date.	Derived from 100092	
ADJUSTMENT VARIABLES – MAIN ANALYSIS				
Age	Baseline	Attained age was the underlying timescale in survival analyses; participants entered the study at the end of accelerometer wear.	Derived from 90011, 34, 52	
Sex	Baseline		31	Male, Female
Ethnicity	Baseline		Derived from 21000	White, non-White
Smoking status	Baseline		Derived from 20116, 3456	Never, previous, current unknown cigarettes/day, current < 15 cigarettes per day, or current ≥ 15 cigarettes per day
Alcohol consumption	Baseline		Derived from 1558	Never, <3 times per week, or ≥ 3 times per week
Education	Baseline		Derived from 6138	School leaver, Further education (education beyond O-Levels/CSEs, excluding college/ university degree) and Higher education (college/university degree)
Townsend Deprivation Index	Baseline	Townsend Deprivation Index of address at time of UKB baseline assessment.	Derived from 189	Divided by quintile of the UK population based on the 2011 UK census[a]
Parity	Baseline	Among females only	Derived from 2734	0, 1-2, 3+, Missing
Use of hormone replacement therapy	Baseline	Among females only	Derived from 2814	No, Yes, Missing

Characteristic	Source	Notes	UK Biobank field	Coding Notes
Use of oral contraceptives pill	Baseline	Among females only	Derived from 2784	No, Yes, Missing
Menopausal status	Baseline	Among females only	Derived from 2724	No, Yes, Not sure or Missing
ADJUSTMENT VARIABLES – SENSITIVITY ANALYSIS				
Body mass index	Baseline		Derived from 21001	For descriptive analyses: Underweight/Normal weight (≤24.9 kilograms/meters^2), Overweight (25-29.9 kilograms/meters^2), Obese (30+ kilograms/meters^2)
Frequency of red and processed meat consumption	Baseline		Derived from 1369, 1379, 1389, 1349	<1, 1–2.9, 3–4.9, 5+ times/week, Missing
Frequency of fresh fruit and vegetable consumption	Baseline		Derived from 1289, 1299, 1309	<3, 3–4.9, 5–7.9, 8+ servings/day, Missing
DESCRIPTIVE VARIABLES – DESCRIPTIVE TABLES				
Self-reported usual Walking pace			924	Slow pace, Steady average pace, Brisk pace, None of the above
Self-rated overall health			2178	Excellent, Good, Fair, Poor
Wear season			Derived from 90001	Winter, Spring, Summer, Autumn

[a] Deprivation based on the 2011 UK census (2011 UK Townsend Deprivation Scores - UK Data Service CKAN. Accessed June 28, 2023. https://statistics.ukdataservice.ac.uk/dataset/2011-uk-townsend-deprivation-scores)

eTable 2. Coding and number of site-specific cancer incident cases in UK Biobank participants.

Cancer site	Cancer registry ICD-10 (if applicable, ICD-O-3)[e]	Death registry ICD-10 codes
**Physical-activity-related cancers**		
Bladder	C670-679	C670-679
Breast[a]	C500-509	C500-509
Colon	C180-189	C180-189
Endometrial[b]	C540-549 and C559	C540-549 and C559
Gastric cardia	C160	C160
Head and neck	C000-009 C019-029, C079-C089, C040-049, C030-039, C050-059, C060-069, C110-119 C090-099, C100-109, C129, C130-139, C140, C142, C148, C320-329	C000-009 C019-029, C079-C089, C040-049, C030-039, C050-059, C060-069, C110-119 C090-099, C100-109, C129, C130-139, C140, C142, C148, C320-329
Kidney	C640-659	C640-659
Liver	C220-229	C220-229
Lung	C340-349	C340-349
Myeloid leukaemia	Any histology of 9840, 9861, 9865-9867, 9869, 9871-9874, 9895-9897, 9898, 9910-9911,9920, 9891, 9863, 9875-9876, 9945-9946, 9860, 9930	Histology based
Myeloma	C880-889, C900-909[f]	C880-889, C900-909
Oesophageal adenocarcinoma	C150-159 & histologies: 8140, 8142, 8144, 8261, 8310, 8480, 8481, 8570	Histology based
Rectal	C190-209	C190-209
**Other site-specific cancers**		
Anal	C210-219	C210-219
Brain	C710-719	C710-719
Cervix[a]	C530-539	C530-539
Gallbladder	C230-239	C230-239
Gastric non-cardia	C161-169	C161-169
Hodgkin lymphoma	C810-819[f]	C810-819
Melanoma skin	C430-439, only histologies: 8720-8790	C430-439

Cancer site	Cancer registry ICD-10 (if applicable, ICD-O-3)[e]	Death registry ICD-10 codes
Non-Hodgkin lymphoma	C820-869, C911, or any histologies of 9823, 9670	C820-869, C911
Chronic lymphocytic leukaemia	C911	C911
Other leukaemia	C910-959, excluding cases classified as Myeloid leukaemia and non-Hodgkin lymphoma[f]	C910-959, excluding cases classified as Myeloid leukaemia and non-Hodgkin lymphoma[f]
Oesophageal squamous cell carcinoma	C150-159, only histologies: 8041,8070,8071,8072,8074	Histology based
Ovary[c]	C560-569	C560-569
Pancreas	C250-259	C250-259
Prostate[d]	C610-619	C610-619
Small intestine	C170-179	C170-179
Thyroid	C730-739	C730-739

[a]Among females, [b]Among females with no history of hysterectomy, [c]Among females with no history of bilateral oophorectomy, [d]Among males.

[e]Unless stated, definitions exclude ICD-O-3 histology codes 9050-9055, 9140, 9590-9992 cell types, which includes Kaposi sarcoma (9140), mesothelioma (9050–9055), lymphomas, leukaemias, myelomas, lymphoreticular, and immunoproliferative diseases (9050-9055, 9140, 9590-9992).

[f]Unless stated, definitions exclude ICD-O-3 histology codes 9050-9055 and 9140, which includes Kaposi sarcoma (9140) and mesothelioma (9050–9055).

eTable 3. Tabulation of number of incident primary events per cancer site in 86 556 UK Biobank participants.

Cancer type	Overall N=86 556	Male N=38 078	Female N=48 478
**Physical-activity-related cancers**	2 669	898	1 771
Bladder	109	86	23
Breast[a]	995	NA	995
Colon	381	207	174
Endometrial[b]	153	NA	153
Gastric cardia	22	18	<5[a]
Head and neck	88	68	20
Kidney	141	83	58
Liver	60	34	26
Lung	295	148	147
Myeloid leukaemia	43	18	25
Myeloma	107	61	46
Oesophageal adenocarcinoma	68	56	12
Rectal	209	121	88
**Other site-specific cancers**			
Anal	26	6	20
Brain	79	42	37
Cervix[a]	12	NA	12
Gallbladder	10	6	<5[a]
Gastric non-cardia	40	25	15
Hodgkin lymphoma	12	7	5
Melanoma skin	328	181	147
Non-Hodgkin lymphoma	316	176	140
Chronic lymphocytic leukaemia	76	49	27
Oesophageal squamous cell carcinoma	29	12	17
Other leukaemia	27	18	9
Ovary[c]	66	NA	66
Pancreas	145	80	65
Prostate[d]	1380	1380	NA
Small intestine	30	14	16
Thyroid	43	15	28

Physical-activity-related cancer was defined as 13 site specific cancers (oesophageal adenocarcinoma, liver, lung, kidney, gastric cardia, endometrial, myeloid leukaemia, myeloma, colon, head and neck, rectal, bladder, and breast). [a]Among females, [b]Among females with no history of hysterectomy, [c]Among males, [d]Among females with no history of bilateral oophorectomy. [e]UK Biobank policy states that tables should have a minimum number of 5 reported participants within a cell.

eTable 4. Strengthening the Reporting of Observational Studies in Epidemiology (STROBE) statement.

	Item No.	Recommendation	Page No.
**Title and abstract**	1	(*a*) Indicate the study's design with a commonly used term in the title or the abstract	1, 3
		(*b*) Provide in the abstract an informative and balanced summary of what was done and what was found	3
**Introduction**			
Background/rationale	2	Explain the scientific background and rationale for the investigation being reported	5-6
Objectives	3	State specific objectives, including any prespecified hypotheses	5-6
**Methods**			
Study design	4	Present key elements of study design early in the paper	5-9
Setting	5	Describe the setting, locations, and relevant dates, including periods of recruitment, exposure, follow-up, and data collection	6-7
Participants	6	(*a*) *Cohort study*—Give the eligibility criteria, and the sources and methods of selection of participants. Describe methods of follow-up<br>*Case-control study*—Give the eligibility criteria, and the sources and methods of case ascertainment and control selection. Give the rationale for the choice of cases and controls<br>*Cross-sectional study*—Give the eligibility criteria, and the sources and methods of selection of participants	6-8
		(*b*) *Cohort study*—For matched studies, give matching criteria and number of exposed and unexposed<br>*Case-control study*—For matched studies, give matching criteria and the number of controls per case	NA
Variables	7	Clearly define all outcomes, exposures, predictors, potential confounders, and effect modifiers. Give diagnostic criteria, if applicable	6-9, eTable 1, eTable 2
Data sources/ measurement	8*	For each variable of interest, give sources of data and details of methods of assessment (measurement). Describe comparability of assessment methods if there is more than one group	6-9, eTable 1

	Item No.	Recommendation	Page No.
Bias	9	Describe any efforts to address potential sources of bias	9
Study size	10	Explain how the study size was arrived at	6-8, eFigure 1
Quantitative variables	11	Explain how quantitative variables were handled in the analyses. If applicable, describe which groupings were chosen and why	6-9, eTable 1
Statistical methods	12	(*a*) Describe all statistical methods, including those used to control for confounding	8-10
		(*b*) Describe any methods used to examine subgroups and interactions	9
		(*c*) Explain how missing data were addressed	6-9, eTable1, eFigure 1
		(*d*) *Cohort study*—If applicable, explain how loss to follow-up was addressed<br>*Case-control study*—If applicable, explain how matching of cases and controls was addressed<br>*Cross-sectional study*—If applicable, describe analytical methods taking account of sampling strategy	8, eFigure 1
		(*e*) Describe any sensitivity analyses	9
Participants	13*	(a) Report numbers of individuals at each stage of study—eg numbers potentially eligible, examined for eligibility, confirmed eligible, included in the study, completing follow-up, and analysed	eFigure 1
		(b) Give reasons for non-participation at each stage	eFigure 1
		(c) Consider use of a flow diagram	eFigure 1
Descriptive data	14*	(a) Give characteristics of study participants (eg demographic, clinical, social) and information on exposures and potential confounders	Table 1
		(b) Indicate number of participants with missing data for each variable of interest	Table 1
		(c) *Cohort study*—Summarise follow-up time (eg, average and total amount)	10
Outcome data	15*	*Cohort study*—Report numbers of outcome events or summary measures over time	10, eTable 3
		Case-control study—Report numbers in each exposure category, or summary measures of exposure	*NA*
		Cross-sectional study—Report numbers of outcome events or summary measures	*NA*

	Item No.	Recommendation	Page No.
Main results	16	(*a*) Give unadjusted estimates and, if applicable, confounder-adjusted estimates and their precision (eg, 95% confidence interval). Make clear which confounders were adjusted for and why they were included	Table 2, eTables 5-13.
		(*b*) Report category boundaries when continuous variables were categorized	eTable 6, eTable 12, eTable 13
		(*c*) If relevant, consider translating estimates of relative risk into absolute risk for a meaningful time period	NA
Other analyses	17	Report other analyses done—eg analyses of subgroups and interactions, and sensitivity analyses	12-13
**Discussion**			
Key results	18	Summarise key results with reference to study objectives	10-12
Limitations	19	Discuss limitations of the study, taking into account sources of potential bias or imprecision. Discuss both direction and magnitude of any potential bias	15-16
Interpretation	20	Give a cautious overall interpretation of results considering objectives, limitations, multiplicity of analyses, results from similar studies, and other relevant evidence	16
Generalisability	21	Discuss the generalisability (external validity) of the study results	15-16
**Other information**			
Funding	22	Give the source of funding and the role of the funders for the present study and, if applicable, for the original study on which the present article is based	19-20

eTable 5. Sequential model adjustments for total physical activity (milligravity units) and risk of incident cancer in 86 556 UK Biobank participants.

		Model 1	Model 2	Model 3
**Cancer type**	**Case count**	**HR per 1 SD (95% CI)**	**HR per 1 SD (95% CI)**	**HR per 1 SD (95% CI)**
Physical-activity-related cancer	2 669	0.85 (0.81-0.88)	0.85 (0.81-0.89)	0.88 (0.84-0.92)
Bladder	109	0.63 (0.50-0.80)	0.69 (0.55-0.87)	0.71 (0.56-0.90)
Breast	995	0.90 (0.85-0.97)	0.91 (0.85-0.97)	0.91 (0.85-0.98)
Colon	381	0.84 (0.75-0.94)	0.84 (0.75-0.95)	0.88 (0.78-0.99)
Endometrial	153	0.76 (0.63-0.91)	0.78 (0.65-0.93)	0.89 (0.74-1.07)
Gastric cardia	22	0.31 (0.17-0.56)	0.39 (0.22-0.70)	0.47 (0.25-0.85)
Head and neck	88	0.75 (0.59-0.97)	0.84 (0.66-1.07)	0.82 (0.64-1.04)
Kidney	141	0.80 (0.66-0.98)	0.86 (0.71-1.04)	0.89 (0.73-1.08)
Liver	60	0.62 (0.45-0.86)	0.65 (0.47-0.90)	0.72 (0.52-1.00)
Lung	295	0.64 (0.55-0.74)	0.75 (0.65-0.86)	0.77 (0.66-0.89)
Myeloid Leukaemia	43	0.95 (0.68-1.33)	0.97 (0.78-1.19)	1.00 (0.81-1.24)
Myeloma	107	0.94 (0.76-1.16)	0.97 (0.70-1.36)	1.03 (0.74-1.45)
Oesophageal adenocarcinoma	68	0.78 (0.59-1.04)	0.89 (0.68-1.16)	1.01 (0.77-1.33)
Rectal	209	0.96 (0.83-1.12)	0.99 (0.85-1.14)	1.00 (0.86-1.16)
Melanoma skin	328	0.90 (0.79-1.01)	0.89 (0.79-1.01)	0.90 (0.80-1.03)
Non-Hodgkin lymphoma	316	0.85 (0.75-0.96)	0.85 (0.75-0.97)	0.87 (0.76-0.99)
Pancreas	145	0.84 (0.69-1.01)	0.86 (0.71-1.04)	0.90 (0.74-1.09)
Prostate	1 380	1.05 (0.99-1.11)	1.04 (0.98-1.10)	1.03 (0.97-1.10)

The standard deviation (SD) of physical activity was 8.3 milligravity unit in the main analytical sample. Hazard ratios (HR) and 95% confidence intervals (CI) were estimated using a Cox proportional hazards model. Models used age as the underlying time variable. Model 1: Unadjusted. Model 2: adjusted for sex, ethnicity, smoking status, alcohol consumption, deprivation, and education. Model 3: Model 2 + adjusted for body mass index. For breast cancer and endometrial cancer, Model 2 and Model 3 were adjusted for ever use of oral contraception, ever use of hormone replacement therapy, menopausal status, and parity. Participants were limited to males for prostate cancer (N=38 078), females for breast cancer (N=48 478), and females without a history of hysterectomy for endometrial cancer (N=41 010).

eTable 6. Sequential model adjustments for quintile of total physical activity (milligravity units), quintile of median daily step count, and risk of incident physical-activity-related cancer in 86 556 UK Biobank participants.

			Model 1	Model 2	Model 3	Model 4
**Quintile of total daily physical activity**	**Total**	**Case count**	**HR (95% CI)**	**HR (95% CI)**	**HR (95% CI)**	**HR (95% CI)**
Q1: <21.6	17 311	718	REF	REF	REF	REF
Q2: 21.6-25.4	17 311	569	0.84 (0.75-0.93)	0.84 (0.75-0.93)	0.86 (0.77-0.96)	0.84 (0.75-0.94)
Q3: 25.5-29.1	17 311	522	0.80 (0.71-0.89)	0.79 (0.71-0.89)	0.83 (0.74-0.93)	0.80 (0.71-0.90)
Q4: 29.2-34.2	17 311	460	0.74 (0.65-0.83)	0.74 (0.65-0.83)	0.78 (0.69-0.88)	0.75 (0.66-0.84)
Q5: 34.3+	17 312	400	0.69 (0.61-0.78)	0.70 (0.61-0.79)	0.76 (0.66-0.86)	0.71 (0.62-0.80)
P value for linear trend			<0.001	<0.001	<0.001	<0.001
**Quintile of daily step count**	**Total**	**Case count**	**HR (95% CI)**	**HR (95% CI)**	**HR (95% CI)**	**HR (95% CI)**
Q1: < 6 319	17 308	687	REF	REF	REF	REF
Q2: 6 319-8 187	17 302	542	0.80 (0.71-0.90)	0.82 (0.74-0.92)	0.85 (0.76-0.95)	0.85 (0.74-0.98)
Q3: 8 188-10 009	17 315	513	0.76 (0.68-0.85)	0.79 (0.71-0.89)	0.83 (0.74-0.93)	0.83 (0.72-0.95)
Q4: 10 010-12 415	17 318	494	0.74 (0.66-0.83)	0.78 (0.69-0.88)	0.83 (0.73-0.93)	0.79 (0.69-0.91)
Q5: 12 416+	17 313	433	0.66 (0.59-0.75)	0.71 (0.63-0.80)	0.76 (0.67-0.86)	0.76 (0.66-0.88)
P value for linear trend			<0.001	<0.001	<0.001	<0.001

Hazard ratios (HR) and 95% confidence intervals (CI) were estimated using a Cox proportional hazards model. Physical-activity-related cancer was defined as 13 site specific cancers (oesophageal adenocarcinoma, liver, lung, kidney, gastric cardia, endometrial, myeloid leukaemia, myeloma, colon, head and neck, rectal, bladder, and breast). Models used age as the underlying time variable. Model 1: Unadjusted. Model 2: adjusted for sex, ethnicity, smoking status, alcohol consumption, deprivation, and education. Model 3: Model 2 + adjusted for body mass index. Model 4: Model 2 + adjusted for fruit and vegetable intake, and red and processed meat intake. REF = reference.

eTable 7. Changes in behaviour time and physical-activity-related cancer risk in 86 556 UK Biobank participants.

	Model 1	Model 2	Model 3	Model 4
**Change in behaviour time**	**HR (95% CI)**	**HR (95% CI)**	**HR (95% CI)**	**HR (95% CI)**
1 hour/day to LIPA from SB	0.97 (0.95-1.00)	0.94 (0.92-0.96)	0.95 (0.93-0.97)	0.94 (0.92-0.96)
1 hour/day to MVPA from SB	0.87 (0.84-0.90)	0.92 (0.89-0.95)	0.94 (0.91-0.98)	0.92 (0.89-0.95)
1 hr/day to Any PA from SB	0.94 (0.93-0.96)	0.93 (0.91-0.95)	0.94 (0.92-0.96)	0.93 (0.91-0.95)
1 hr/day to MVPA from LIPA	0.90 (0.86-0.93)	0.99 (0.94-1.03)	1.00 (0.96-1.05)	0.99 (0.94-1.03)

Hazard ratios (HR) and 95% confidence intervals (CI for incident physical-activity-related cancer associated with reallocating time to from one behaviour to another behaviour. Models are based on 2 669 events in 86 556 participants. HRs are relative to the mean behaviour composition (8.1 hours/day sleep, 10.5 hours/day sedentary behaviour (SB), 4.9 hours/day light intensity physical activity behaviours (LIPA), 0.46 hours/day (27.4 minutes/day) moderate-vigorous intensity physical (MVPA) activity behaviours). The “Any PA” category represents time in either LIPA or MVPA and was generated using a combined category for LIPA and MVPA activity time. Physical-activity-related cancer was defined as 13 site specific cancers (oesophageal adenocarcinoma, liver, lung, kidney, gastric cardia, endometrial, myeloid leukaemia, myeloma, colon, head and neck, rectal, bladder, and breast). Models used age as the underlying time variable. Model 1: Unadjusted. Model 2: adjusted for sex, ethnicity, smoking status, alcohol consumption, deprivation, and education. Model 3: Model 2 + adjusted for body mass index. Model 4: Model 2 + adjusted for fruit and vegetable intake, and red and processed meat intake. Models are based on 2 669 events in 86 556 participants.

eTable 8. Sequential model adjustments for median daily step count and physical-activity-related cancer risk in 86 556 UK Biobank participants.

	Model 1	Model 2	Model 3	Model 4	Model 5
**Median daily steps**	**HR (95% CI)**	**HR (95% CI)**	**HR (95% CI)**	**HR (95% CI)**	**HR (95% CI)**
3 000	1.27 (1.11 - 1.45)	1.22 (1.07 - 1.40)	1.19 (1.04 - 1.36)	1.22 (1.07 - 1.39)	1.22 (1.04 - 1.44)
5 000 (REF, ~10th percentile)	1.00 (1.00 - 1.00)	1.00 (1.00 - 1.00)	1.00 (1.00 - 1.00)	1.00 (1.00 - 1.00)	1.00 (1.00 - 1.00)
7 000	0.86 (0.80 - 0.92)	0.88 (0.82 - 0.94)	0.90 (0.83 - 0.96)	0.88 (0.82 - 0.94)	0.88 (0.80 - 0.96)
9 000	0.79 (0.71 - 0.87)	0.82 (0.74 - 0.90)	0.85 (0.77 - 0.94)	0.82 (0.74 - 0.91)	0.82 (0.73 - 0.93)
11 000	0.76 (0.68 - 0.84)	0.79 (0.71 - 0.88)	0.84 (0.75 - 0.93)	0.80 (0.72 - 0.89)	0.79 (0.70 - 0.90)
13 000	0.73 (0.65 - 0.82)	0.77 (0.69 - 0.86)	0.82 (0.73 - 0.92)	0.78 (0.69 - 0.87)	0.78 (0.67 - 0.89)
16 000 (~95th percentile)	0.66 (0.57 - 0.77)	0.71 (0.61 - 0.82)	0.76 (0.65 - 0.88)	0.72 (0.61 - 0.83)	0.75 (0.62 - 0.90)
P value for linear trend	<0.001	<0.001	<0.001	<0.001	<0.001

Estimated hazard ratios (HR) and 95% confidence intervals (CI) were computed using a Cox proportional hazards model with restricted cubic spline functions. Observations were trimmed at the 1% and 99% of the distribution and 3 knots were placed at the 5th, 50th, and 95th percentile for the exposures in the main sample. The ~10th percentile was set as the referent group (5 000 steps). Physical-activity-related cancer was defined as 13 site specific cancers (oesophageal adenocarcinoma, liver, lung, kidney, gastric cardia, endometrial, myeloid leukaemia, myeloma, colon, head and neck, rectal, bladder, and breast). Models used age as the underlying time variable. Model 1: Unadjusted. Model 2: adjusted for sex, ethnicity, smoking status, alcohol consumption, deprivation, and education. Model 3: Model 2 + adjusted for body mass index. Model 4 + adjusted for fruit and vegetable intake, and red and processed meat intake. Model 5: Model 2 with 2-year exclusion, N=81 790. REF = reference.

eTable 9. Sequential model adjustments for total physical activity (milligravity units) and risk of incident cancer in the UK Biobank among 38 078 male UK Biobank participants.

		Model 1	Model 2	Model 3
**Cancer type**	**Case count**	**HR per 1 SD (95% CI)**	**HR per 1 SD (95% CI)**	**HR per 1 SD (95% CI)**
Physical-activity-related cancer	898	0.77 (0.71-0.84)	0.80 (0.74-0.87)	0.84 (0.78-0.91)
Bladder	86	0.70 (0.54-0.92)	0.72 (0.56-0.95)	0.76 (0.58-1.00)
Breast	NA	NA	NA	NA
Colon	207	0.77 (0.65-0.91)	0.76 (0.64-0.90)	0.80 (0.67-0.95)
Endometrial	NA	NA	NA	NA
Gastric cardia	18	0.34 (0.17-0.67)	0.41 (0.21-0.79)	0.49 (0.25-0.98)
Head and neck	68	0.86 (0.65-1.13)	0.91 (0.69-1.18)	0.88 (0.67-1.16)
Kidney	83	0.85 (0.66-1.09)	0.89 (0.69-1.13)	0.90 (0.70-1.16)
Liver	34	0.76 (0.50-1.15)	0.77 (0.51-1.17)	0.84 (0.55-1.29)
Lung	148	0.54 (0.43-0.68)	0.64 (0.52-0.80)	0.68 (0.54-0.84)
Myeloid Leukaemia	18	1.04 (0.63-1.72)	1.11 (0.67-1.82)	1.26 (0.76-2.10)
Myeloma	61	0.92 (0.69-1.22)	0.93 (0.70-1.24)	0.94 (0.70-1.27)
Oesophageal adenocarcinoma	56	0.83 (0.61-1.13)	0.89 (0.66-1.20)	1.01 (0.74-1.38)
Rectal	121	0.95 (0.78-1.16)	0.96 (0.79-1.17)	0.98 (0.80-1.20)
Melanoma skin	181	0.90 (0.77-1.07)	0.88 (0.75-1.05)	0.90 (0.76-1.08)
Non-Hodgkin lymphoma	176	0.91 (0.77-1.08)	0.90 (0.75-1.07)	0.91 (0.76-1.09)
Pancreas	80	0.84 (0.64-1.09)	0.86 (0.66-1.11)	0.93 (0.71-1.21)
Prostate	1 380	1.05 (0.99-1.11)	1.04 (0.98-1.10)	1.03 (0.97-1.10)

The standard deviation (SD) of physical activity was 8.3 milligravity unit in the main analytical sample. Hazard ratios (HR) and 95% confidence intervals (CI) were estimated using a Cox proportional hazards model. Models used age as the underlying time variable. Model 1: Unadjusted. Model 2: adjusted for ethnicity, smoking status, alcohol consumption, deprivation, and education. Model 3: Model 2 + adjusted for body mass index.

eTable 10. Sequential model adjustments for total physical activity (milligravity units) and risk of incident cancer in the UK Biobank among 48 478 female UK Biobank participants.

		Model 1	Model 2	Model 3
**Cancer type**	**Case count**	**HR per 1 SD (95% CI)**	**HR per 1 SD (95% CI)**	**HR per 1 SD (95% CI)**
Physical-activity-related cancer	1 771	0.87 (0.83-0.92)	0.88 (0.84-0.93)	0.90 (0.86-0.95)
Bladder	23	0.56 (0.33-0.94)	0.54 (0.31-0.91)	0.52 (0.30-0.90)
Breast	995	0.90 (0.85-0.97)	0.91 (0.85-0.97)	0.91 (0.85-0.98)
Colon	174	0.95 (0.80-1.12)	0.94 (0.80-1.11)	0.96 (0.81-1.14)
Endometrial	153	0.76 (0.63-0.91)	0.78 (0.65-0.93)	0.89 (0.74-1.07)
Gastric cardia	<5[a]	NA	NA	NA
Head and neck	20	0.55 (0.32-0.96)	0.61 (0.36-1.05)	0.59 (0.34-1.04)
Kidney	58	0.78 (0.58-1.06)	0.82 (0.61-1.11)	0.88 (0.64-1.19)
Liver	26	0.48 (0.29-0.80)	0.50 (0.30-0.84)	0.55 (0.32-0.93)
Lung	147	0.75 (0.62-0.91)	0.86 (0.71-1.04)	0.87 (0.72-1.06)
Myeloid Leukaemia	25	0.89 (0.57-1.38)	0.89 (0.57-1.39)	0.90 (0.57-1.42)
Myeloma	46	0.99 (0.72-1.36)	1.01 (0.74-1.39)	1.08 (0.79-1.49)
Oesophageal adenocarcinoma	12	0.80 (0.41-1.55)	0.89 (0.46-1.72)	1.02 (0.51-2.01)
Rectal	88	1.02 (0.82-1.27)	1.02 (0.82-1.28)	1.03 (0.82-1.29)
Melanoma skin	147	0.92 (0.77-1.09)	0.90 (0.75-1.08)	0.90 (0.75-1.09)
Non-Hodgkin lymphoma	140	0.80 (0.66-0.97)	0.81 (0.67-0.98)	0.82 (0.67-1.00)
Pancreas	65	0.86 (0.65-1.14)	0.88 (0.66-1.17)	0.87 (0.65-1.17)
Prostate	NA	NA	NA	NA

The standard deviation (SD) of physical activity was 8.3 milligravity unit in the main analytical sample. Hazard ratios (HR) and 95% confidence intervals (CI) were estimated using a Cox proportional hazards model. Models used age as the underlying time variable. Model 1: Unadjusted. Model 2: Adjusted for ethnicity, smoking status, alcohol consumption, deprivation, education, ever use of oral contraception, ever use of hormone replacement therapy, menopausal status, and parity. Model 3: Model 2 + adjusted for body mass index. Participants were limited to females without a history of hysterectomy for endometrial cancer, N=41 010. [a]UK Biobank policy states that tables should have a minimum number of 5 reported participants within a cell.

eTable 11. Sex-specific adjusted hazard ratios for median daily step count and physical-activity-related cancer risk in 38 078 male UK Biobank participants and 48 478 female UK Biobank participants.

	**Male**	**Female**
**Median daily steps**	**HR (95% CI)**	**HR (95% CI)**
3 000	1.42 (1.15 - 1.75)	1.11 (0.93 - 1.33)
5 000 (REF, ~10th percentile)	1.00 (1.00 - 1.00)	1.00 (1.00 - 1.00)
7 000	0.81 (0.72 - 0.91)	0.92 (0.84 - 1.01)
9 000	0.72 (0.61 - 0.85)	0.87 (0.77 - 0.99)
11 000	0.69 (0.57 - 0.82)	0.86 (0.75 - 0.98)
13 000	0.69 (0.57 - 0.84)	0.82 (0.71 - 0.94)
16 000 (~95th percentile)	0.65 (0.50 - 0.84)	0.74 (0.61 - 0.90)
P value for linear trend	<0.001	<0.001

Estimated hazard ratios (HR) and 95% confidence intervals (CI) were computed using a Cox proportional hazards model with restricted cubic spline functions. Observations were trimmed at the 1% and 99% of the distribution and 3 knots were placed at the 5th, 50th, and 95th percentile for the exposures in the main sample. The ~10th percentile was set as the referent group (5 000 steps). Physical-activity-related cancer was defined as 13 site specific cancers (oesophageal adenocarcinoma, liver, lung, kidney, gastric cardia, endometrial, myeloid leukaemia, myeloma, colon, head and neck, rectal, bladder, and breast). Models used attained age as the underlying time variable and were adjusted for ethnicity, smoking status, alcohol consumption, deprivation, and education. The overall model was further adjusted for sex and the female model was further adjusted for ever use of oral contraception, ever use of hormone replacement therapy, menopausal status, and parity. REF = reference.

eTable 12. Models for quintile of total physical activity (milligravity units), quintile of median daily step count, and risk of incident physical-activity-related cancer in UK Biobank participants who were never smokers (N=49 736).

**Quintile of physical activity**	**Total**	**Case count**	**HR (95% CI)**
Q1: <22.0	9 947	335	REF
Q2: 22.0-25.7	9 946	269	0.83 (0.70-0.97)
Q3: 25.8-29.5	9 948	265	0.83 (0.71-0.98)
Q4: 29.6-34.5	9 947	244	0.80 (0.67-0.94)
Q5: 34.6+	9 948	195	0.68 (0.57-0.82)
P value for linear trend			<0.001
**Quintile of daily step count**	**Total**	**Case count**	**HR (95% CI)**
Q1: <6 480	9 941	308	REF
Q2: 6 480-8 318	9 953	281	0.93 (0.79-1.09)
Q3: 8 319-10 109	9 943	253	0.84 (0.71-0.99)
Q4: 10 110-12 469	9 951	255	0.85 (0.72-1.01)
Q5: 12, 470+	9 948	211	0.73 (0.61-0.87)
P value for linear trend			0.001

Physical-activity-related cancer was defined as 13 site specific cancers (oesophageal adenocarcinoma, liver, lung, kidney, gastric cardia, endometrial, myeloid leukaemia, myeloma, colon, head and neck, rectal, bladder, and breast). Hazard ratios (HR) and 95% confidence intervals (CI) were estimated using a Cox proportional hazards model. Models used age as the underlying time variable. Model was adjusted for sex, ethnicity, alcohol consumption, deprivation, and education. REF = reference.

eTable 13. Models for quintile of total physical activity (milligravity units), quintile of median daily step count, and risk of incident physical-activity-related cancer in 86 556 UK Biobank participants before and after removing the first two years of follow-up (N=83 435).

**Quintile of physical activity**	**Total**	**Case count**	**HR (95% CI)**
Q1: <21.7	16 687	480	REF
Q2: 21.7-25.4	16 687	374	0.83 (0.72-0.95)
Q3: 25.5-29.2	16 687	367	0.84 (0.73-0.96)
Q4: 29.3-34.3	16 687	320	0.77 (0.67-0.89)
Q5: 34.4+	16 687	280	0.73 (0.63-0.85)
P value for linear trend			0.001
**Quintile of daily step count**	**Total**	**Case count**	**HR (95% CI)**
Q1: < 6 343	16 687	456	REF
Q2: 6 343-8 205	16 683	371	0.85 (0.74-0.98)
Q3: 8 206-10 021	16 689	353	0.83 (0.72-0.95)
Q4: 10 022-12 433	16 682	333	0.79 (0.69-0.91)
Q5: 12 434+	16 694	308	0.76 (0.66-0.88)
P value for linear trend			<0.001

Hazard ratios (HR) and 95% confidence intervals (CI) were estimated using a Cox proportional hazards model. Physical-activity-related cancer was defined as 13 site specific cancers (oesophageal adenocarcinoma, liver, lung, kidney, gastric cardia, endometrial, myeloid leukaemia, myeloma, colon, head and neck, rectal, bladder, and breast). Models used age as the underlying time variable. Model was adjusted for sex, ethnicity, smoking status, alcohol consumption, deprivation, and education. REF = reference.

eFigure 1. Participant flow diagram for the analysis of daily physical activity and step count measured by accelerometers in UK Biobank participants.

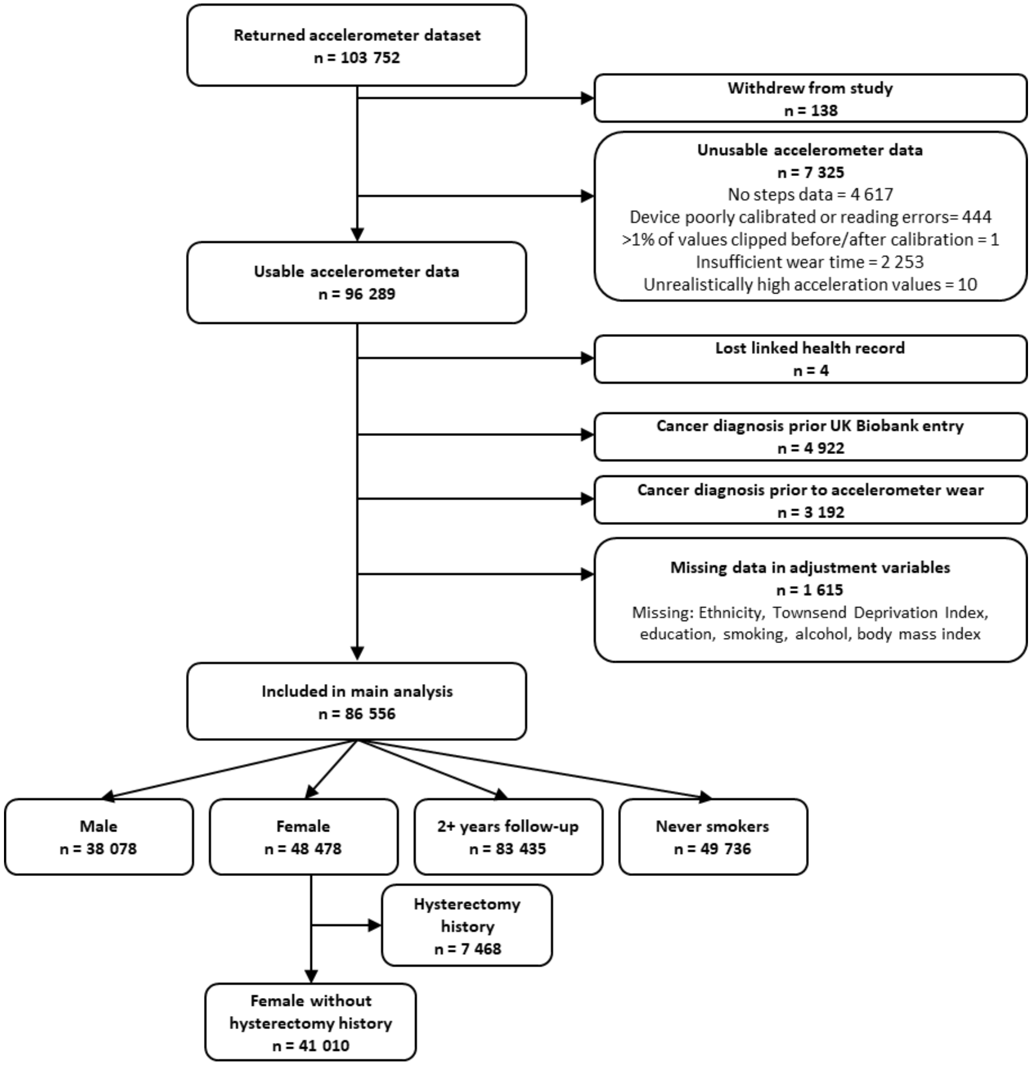

eFigure 2. Hazard Ratios reallocating time to a given movement behaviour from all other behaviours proportionally and incident physical-activity-related cancer in 86 556 UK Biobank participants.

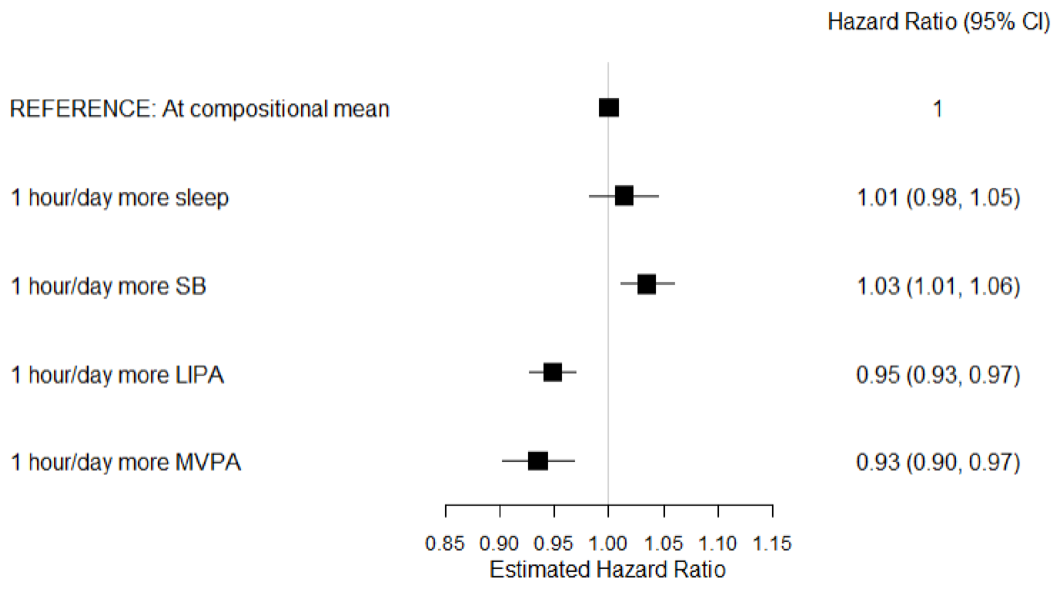

Hazard ratios (HR) and 95% confidence intervals (CI) for incident physical-activity-related cancer associated with reallocating time to from one behaviour to another behaviour proportionally. Models are based on 2 669 events in 86 556 participants. Hazard ratios are relative to the mean behaviour composition (8.1 hours/day sleep, 10.5 hours/day sedentary behaviour (SB), 4.9 hours/day light intensity physical activity behaviours (LIPA), 0.46 hours/day (27.4 minutes/day) moderate-vigorous intensity physical (MVPA) activity behaviours). Models used attained age as the underlying time variable and were adjusted for sex, ethnicity, smoking status, alcohol consumption, deprivation, and education. Models are based on 2 669 events in 86 556 participants.

eFigure 3. Hazard ratios for all behaviour pairs and incident physical-activity-related cancer risk for estimated using a multivariable-adjusted Cox regression model in 86 556 UK Biobank participants before (blue) and after adjusting for body mass index as a sensitivity analysis.

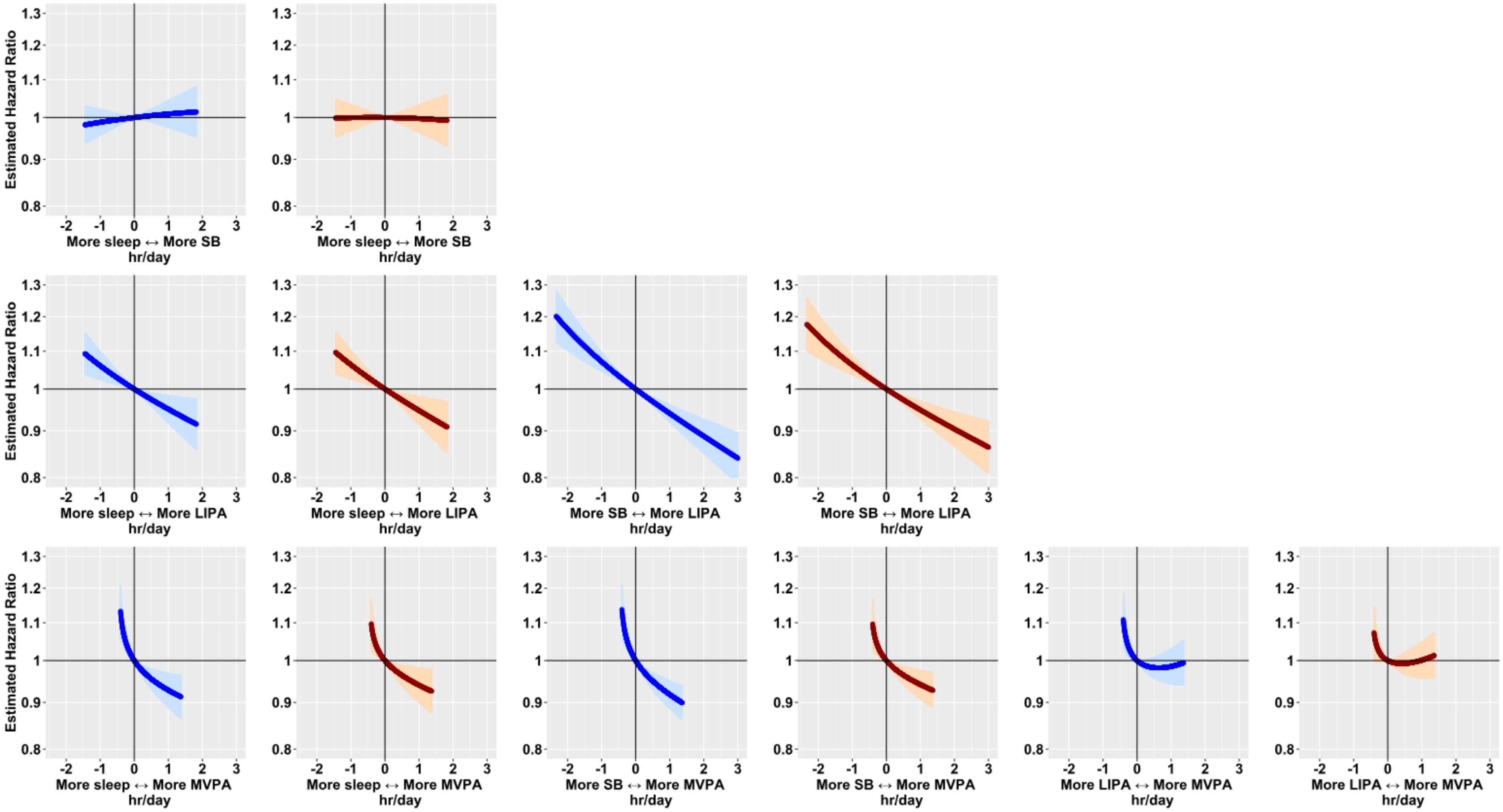

Hazard ratios are relative to the mean behaviour composition (8.1 hours/day sleep, 10.5 hours/day sedentary behaviour, 4.9 hours/day light intensity physical activity behaviours, 0.46 hours/day (27.4 minutes/day) moderate-vigorous intensity physical activity behaviours). Models used attained age as the underlying time variable and were adjusted for sex, ethnicity, smoking status, alcohol consumption, deprivation, and education. Models are based on 2 669 events in 86 556 participants. SB = sedentary behaviour, LIPA = light intensity activity, MVPA = moderate-vigorous intensity activity, hr = hour.

eFigure 4. Hazard ratios for all behaviour pairs and incident physical-activity-related cancer risk for estimated using a multivariable-adjusted Cox regression model in 86 556 UK Biobank participants before (blue) and after adjusting for dietary factors as a sensitivity analysis.

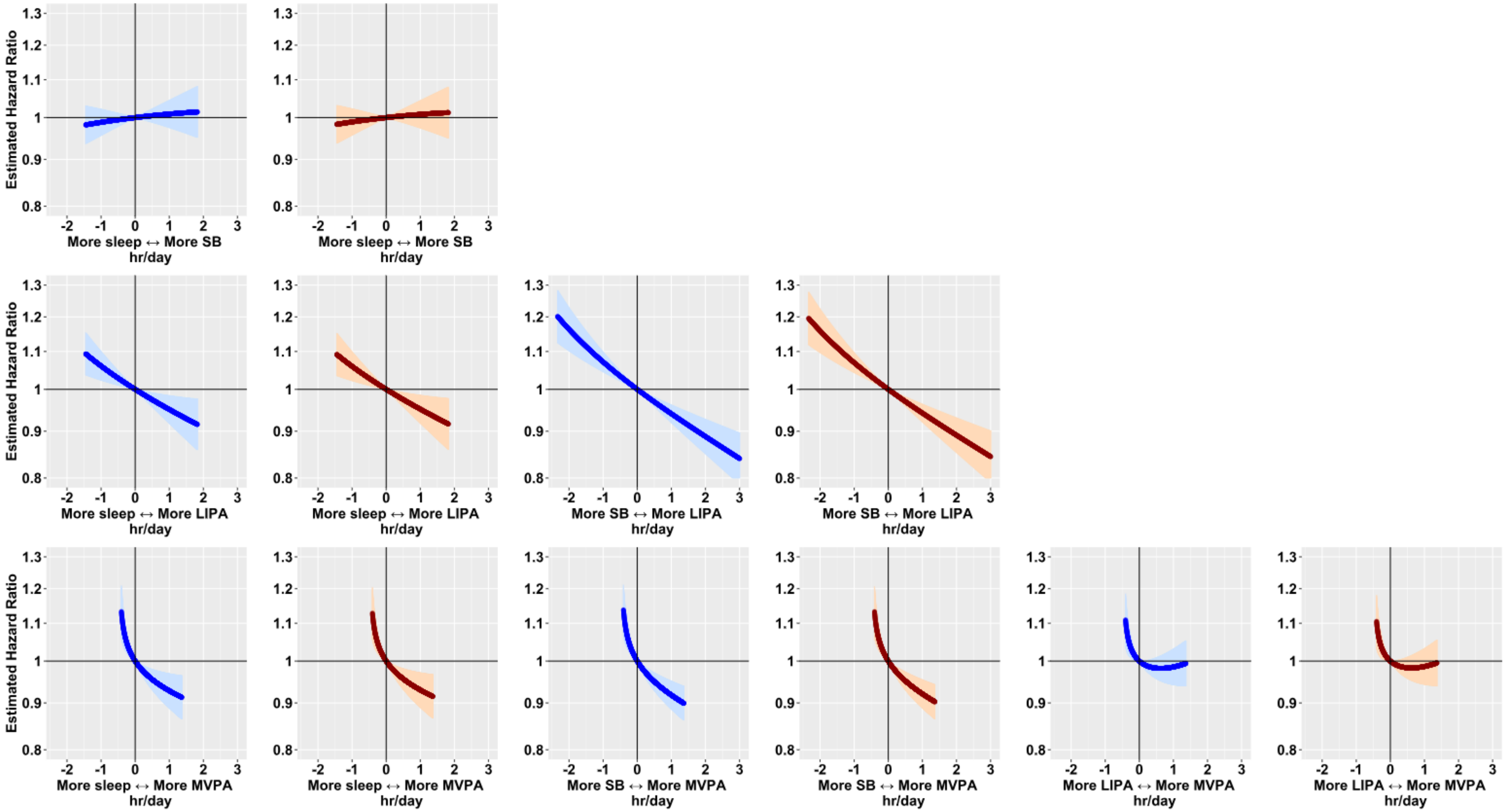

Hazard ratios are relative to the mean behaviour composition (8.1 hours/day sleep, 10.5 hours/day sedentary behaviour, 4.9 hours/day light intensity physical activity behaviours, 0.46 hours/day (27.4 minutes/day) moderate-vigorous intensity physical activity behaviours). Models used attained age as the underlying time variable and were adjusted for sex, ethnicity, smoking status, alcohol consumption, deprivation, and education. Models are based on 2 669 events in 86 556 participants. SB = sedentary behaviour, LIPA = light intensity activity, MVPA = moderate-vigorous intensity activity, hr = hour.

eFigure 5. Hazard ratios for all behaviour pairs and incident physical-activity-related cancer risk for estimated using a multivariable-adjusted Cox regression model among 48 478 female participants (blue) and 38 078 male participants (red).

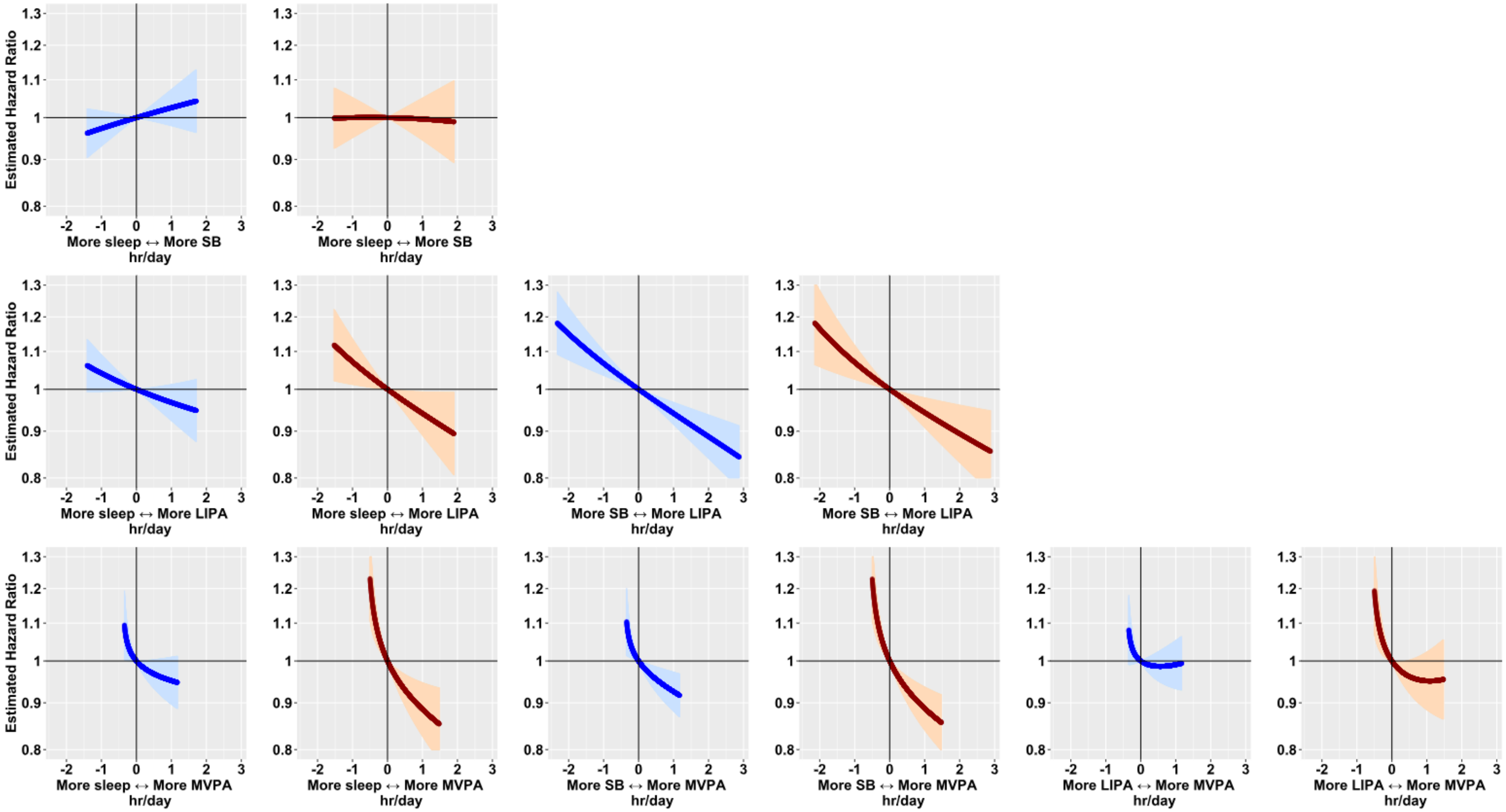

Hazard ratios are relative to the mean behaviour composition in each sex (female – 8.2 hours/day sleep, 10.1 hours/day sedentary behaviour, 5.3 hours/day light intensity physical activity behaviours, 0.38 hours/day (22.8 minutes/day) moderate-vigorous intensity physical activity behaviours; male – 7.9 hours/day sleep, 11.0 hours/day sedentary behaviour, 4.5 hours/day light intensity physical activity behaviours, 0.58 hours/day (35.0 minutes/day) moderate-vigorous intensity physical activity behaviours). Models used attained age as the underlying time variable and were adjusted for ethnicity, smoking status, alcohol consumption, deprivation, and education. Female models were further adjusted for ever use of oral contraception, ever use of hormone replacement therapy, menopausal status, and parity Model is based on 1 771 events in 48 478 female participants and 898 events in 38 078 male participants. SB = sedentary behaviour, LIPA = light intensity activity, MVPA = moderate-vigorous intensity activity, hr = hour.

eFigure 6. Hazard ratios for all behaviour pairs and incident physical-activity-related cancer risk for estimated using a multivariable-adjusted Cox regression model in 86 556 UK Biobank participants before (blue) and after restricting to never smokers (red, N=49 736).

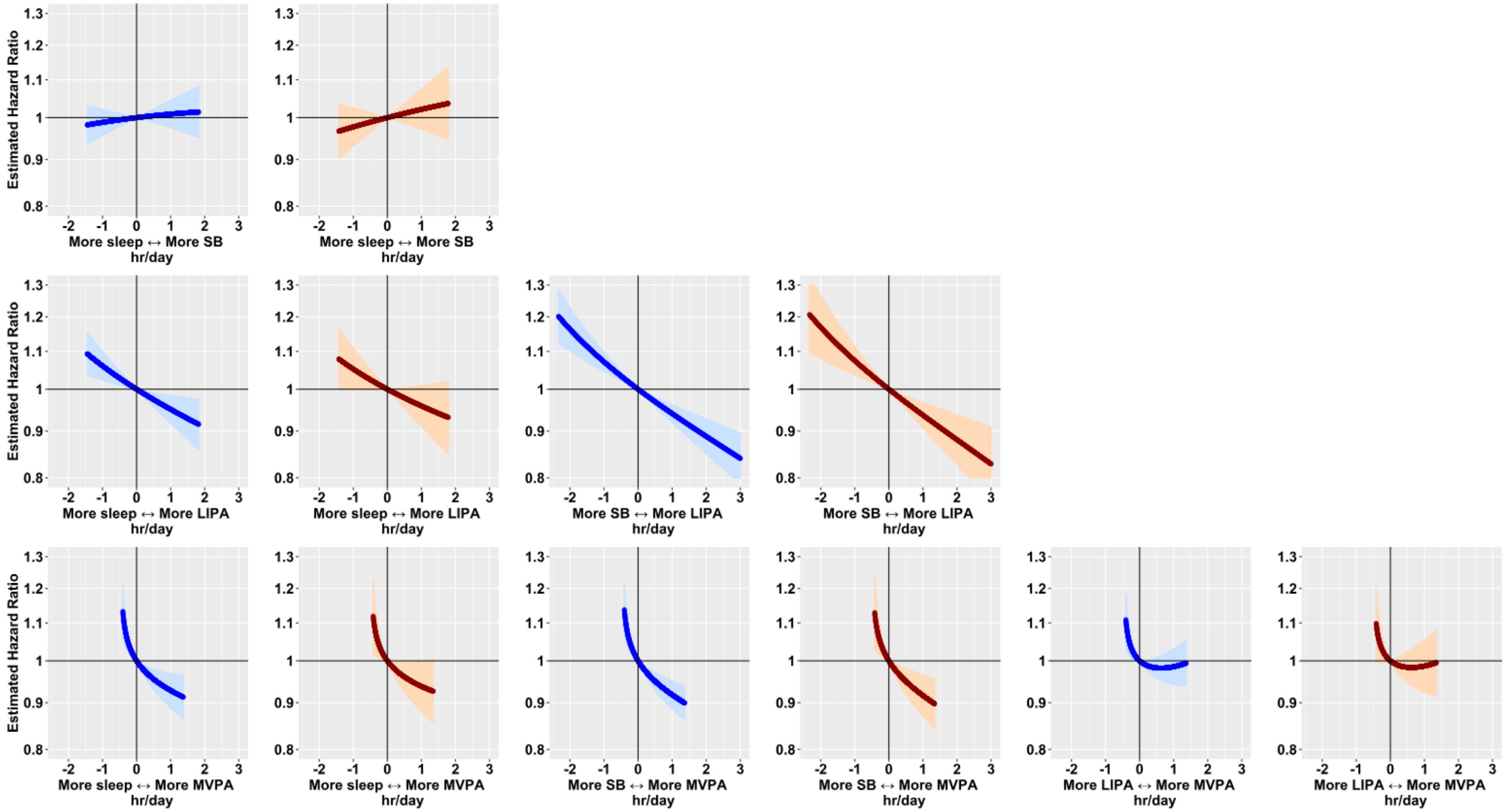

Hazard ratios are relative to the mean behaviour composition in each sample (full sample – 8.1 hours/day sleep, 10.5 hours/day sedentary behaviour, 4.9 hours/day light intensity physical activity behaviours, 0.46 hours/day (27.4 minutes/day) moderate-vigorous intensity physical activity behaviours; Non-smoking subgroup – 8.1 hours/day sleep, 10.4 hours/day sedentary behaviour, 5.0 hours/day light intensity physical activity behaviours, 0.48 hours/day (28.7 minutes/day) moderate-vigorous intensity physical activity behaviours). Models used attained age as the underlying time variable and were adjusted for ethnicity, smoking status, alcohol consumption, deprivation, and education. The main model is based on 2 669 events in 86 556 participants and the sensitivity analysis model is based on 1 308 events in 49 736 participants. SB = sedentary behaviour, LIPA = light intensity activity, MVPA = moderate-vigorous intensity activity, hr = hour.

eFigure 7. Hazard ratios for all behaviour pairs and incident physical-activity-related cancer risk for estimated using a multivariable-adjusted Cox regression model in 86 556 UK Biobank participants before (blue) and after removing the first two years of follow-up (red, N=83 435).

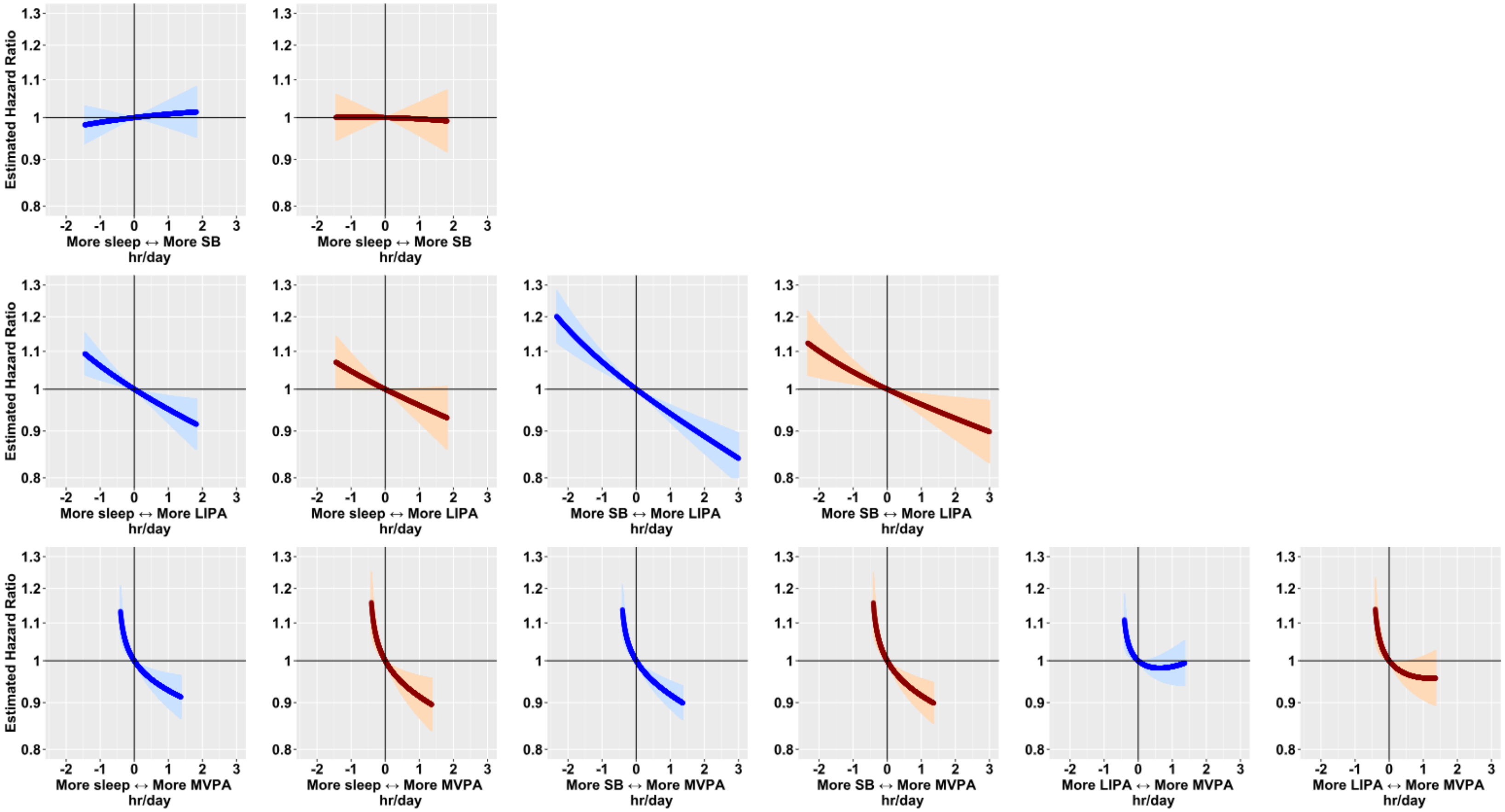

Hazard ratios are relative to the mean behaviour composition in each sample (full sample – 8.1 hours/day sleep, 10.5 hours/day sedentary behaviour, 4.9 hours/day light intensity physical activity behaviours, 0.46 hours/day (27.4 minutes/day) moderate-vigorous intensity physical activity behaviours; sample with at least 2-years follow-up – 8.1 hours/day sleep, 10.5 hours/day sedentary behaviour, 4.9 hours/day light intensity physical activity behaviours, 0.46 hours/day (27.6 minutes/day) moderate-vigorous intensity physical activity behaviours). Models used attained age as the underlying time variable and were adjusted for ethnicity, smoking status, alcohol consumption, deprivation, and education. The main model is based on 2 669 events in 86 556 participants and the sensitivity analysis model is based on 1 821 events in 83 435 participants. SB = sedentary behaviour, LIPA = light intensity activity, MVPA = moderate-vigorous intensity activity, hr = hour.
